# Sirolimus versus mycophenolate mofetil for the treatment of lupus nephritis: Results from a real-world CSTAR cohort study

**DOI:** 10.1515/rir-2025-0011

**Published:** 2025-07-01

**Authors:** Wei Bai, Liying Peng, Yinli Gui, Yunzhuan Chen, Xinwang Duan, Xiaofeng Li, Hongfeng Zhang, Yuehong Huo, Jian Xu, Pingting Yang, Yanhong Wang, Chanyuan Wu, Jiuliang Zhao, Qian Wang, Xiaomei Leng, Xinping Tian, Mengtao Li, Xiaofeng Zeng

**Affiliations:** Department of Rheumatology and Clinical Immunology, Peking Union Medical College Hospital, Chinese Academy of Medical Sciences, Peking Union Medical College, National Clinical Research Center for Dermatologic and Immunologic Diseases (NCRC-DID), Ministry of Science & Technology, State Key Laboratory of Complex Severe and Rare Diseases, Peking Union Medical College Hospital, Key Laboratory of Rheumatology and Clinical Immunology, Ministry of Education, Beijing, China; Department of Rheumatology, People’s Hospital of Zhengzhou, Henan Province, China; Department of Rheumatology, The Second Affiliated Hospital of Nanchang University, Nanchang, Jiangxi Province, China; Department of Rheumatology, Second Hospital of Shanxi Medical University, Taiyuan, Shanxi Province, China; Department of Rheumatology, The First Affiliated Hospital of Dalian Medical University, Dalian, Liaoning Province, China; Department of Rheumatology, The Fifth People’s Hospital of Datong, Shanxi Province, China; Department of Rheumatology, The First Affiliated Hospital of Kunming Medical University, Kunming, Yunnan Province, China; Department of Rheumatology, The First Hospital of China Medical University, Shenyang, Liaoning Province, China; Department of Epidemiology and Bio-statistics (YW), Institute of Basic Medical Science, Chinese Academy of Medical Science & Peking Union Medical College, Beijing 100053, China

**Keywords:** systemic lupus erythematosus, lupus nephritis, sirolimus, LLDAS/remission, T2T strategy

## Abstract

**Background and Objectives:**

No prior studies have directly compared sirolimus with the standard of care (SoC) for lupus nephritis (LN) patients. This study aimed to compare the efficacy and safety of sirolimus with mycophenolate mofetil (MMF) for the treatment of LN.

**Methods:**

A real-world cohort study based on the Chinese SLE Treatment and Research (CSTAR) registry was conducted. Patients with active LN who were prescribed either sirolimus or MMF were enrolled. Propensity score matching was applied to ensure comparable baseline disease conditions. SLE disease activity indices, serological parameters, steroid doses, renal efficacy, and adverse events were evaluated at 3-month, 6-month, and 12-month follow-ups.

**Results:**

Data from 53 patients in each group were analyzed. Sirolimus demonstrated clinical effectiveness comparable to MMF, as evidenced by similar rates of lupus nephritis remission and lupus low disease activity state (LLDAS) /remission or a clinical response (reduction of SLE Disease Activity Index 2000 [SLEDAI-2K] ≥4 and increase in physician’s global assessment [PhGA] < 0.3), as well as changes in 24-hour urine protein level, SLEDAI-2K score, PhGA score, and steroid tapering effect (*P* ≥ 0.05 at all follow-up timepoints). Notably, sirolimus group exhibited greater improvements in complement levels compared to MMF group at 3, 6, and 12 months. Ten adverse events in sirolimus group and one in MMF group were reported, with no severe adverse events.

**Conclusion:**

Sirolimus demonstrated comparable efficacy to MMF in the treatment of LN and glucocorticoid tapering, with additional benefits in serological improvement. Furthermore, sirolimus was well tolerated in LN patients, supporting its potential as a therapeutic option for LN.

## Introduction

Systemic lupus erythematosus (SLE) is a complex autoimmune disease characterized by variable manifestations affecting multiple organ systems, often leading to organ damage and increased mortality.^[1,2]^ Lupus nephritis (LN) occurs in approximately 40% of SLE patients ^[3,4]^ and is associated with a heightened risk of end-stage renal disease (ESRD) and elevated mortality rates.^[2,3,5,6]^

While biological agents such as belimumab ^[7,8]^ and rituximab^[9]^ have shown promise in the treatment of SLE and LN, conventional immunosuppressive therapies, such as cyclophosphamide (CYC), mycophenolate (MMF), methotrexate (MTX), azathioprine (AZA), and calcineurin inhibitors (CNIs) remain the standard of care (SoC) for managing SLE and LN.^[4,10,11]^ Furthermore, the treat-to-target (T2T) strategy has been recommended for SLE in recent years.^[12]^ Achieving remission or a state of lupus low disease activity state (LLDAS) with effective immunosuppressants or biologics has emerged as a key treatment goal and a predictor of outcomes in SLE.^[13, 14, 15]^ Regarding LN, the treatment target focuses on clinical response, particularly primary efficacy renal response, which has been linked to improved prognosis and is widely used as an endpoint in recent clinical trials.^[8]^ Given the severity of LN and the adverse effects associated with current medications, there is a pressing need for novel treatment options and further evidence supporting the T2T approach to improve the prognosis of LN.

Sirolimus, an inhibitor of the mammalian target of rapamycin (mTOR), has been implicated in the pathogenesis of SLE.^[16]^ Emerging studies have demonstrated the efficacy and safety of sirolimus as a novel immunosuppressant for SLE and LN.^[17, 18, 19]^ However, no comparative studies have directly evaluated sirolimus against the SoC for LN patients. Leveraging the Chinese SLE Treatment and Research (CSTAR) group, the largest registry of SLE in China,^[20]^ we conducted a prospective real-world cohort study to compare the effectiveness and safety of sirolimus versus MMF in the treatment of LN.

## Methods

### Patients

**Figure 1:**
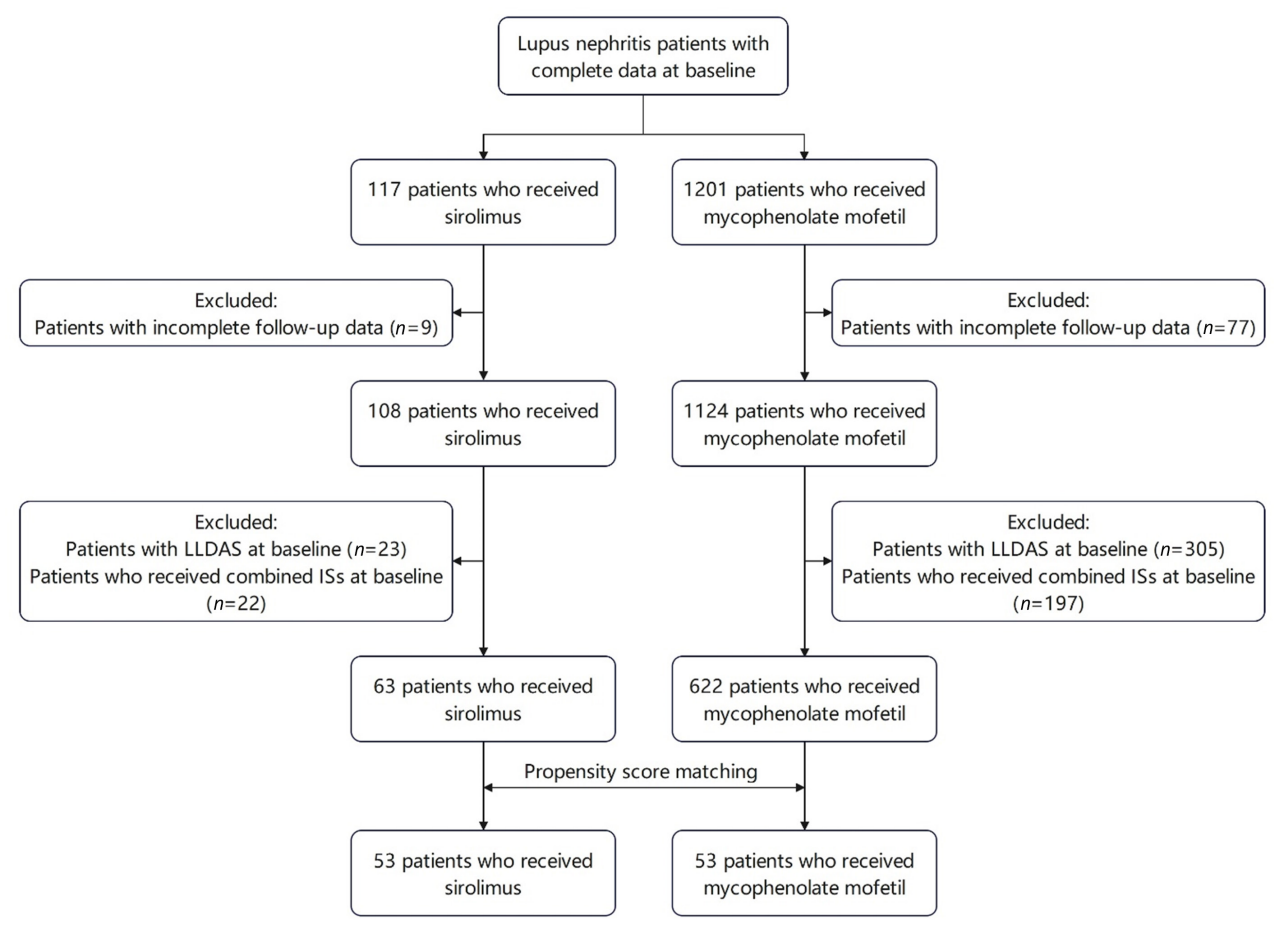
The screening flow chart. LLDAS: lupus low disease activity state.

As of March 2022, the CSTAR registry included 338 rheumatology centers across 31 provinces in China. Based on the CSTAR registry, this prospective real-world study enrolled LN patients who were prescribed either sirolimus or MMF between March 2012 and March 2022. This study was approved by the Medical Ethics Committee of Peking Union Medical College Hospital (PUMCH)(approval number: S-478), and written informed consent was obtained from all recruited patients. Patients were diagnosed with LN based on their fulfilment of the corresponding items for lupus nephritis from the SLE classification criteria established by American College of Rheumatology (ACR) in 1997,^[21]^ the Systemic Lupus International Collaborating Clinics (SLICC)/ACR in 2012,^[22]^ or the European League Against Rheumatism/ACR in 2019.^[1]^ Within the CSTAR cohort, 108 LN patients were treated with sirolimus, and 1124 LN patients were treated with MMF, all of whom had completed baseline and 3- or 6-month follow-up data. Inclusion criteria required patients to have active lupus status and to have been switched to or initiated on sirolimus or MMF alone as immunosuppressant at baseline. The dosage of background immunosuppressants in patients who have been initiated on sirolimus or MMF at baseline remained stable for at least one month before baseline. Patients receiving combined immunosuppressants, including CYC, MTX, AZA, tacrolimus (TAC) or cyclosporin A, in addition to sirolimus or MMF at baseline, or those who had already achieved LLDAS/remission at baseline, were excluded. The screening flowchart is illustrated in Figure 1. After applying the inclusion and exclusion criteria, a total of 63 patients treated with sirolimus and 622 patients treated with MMF were included in this study.

### Data Collection and Endpoint Measures

All CSTAR centers adhered to standardized protocol-directed methods for patient evaluations and data recording. Data collected at baseline, 3 months, 6 months, and 12 months included patients’ demographic information, clinical features, laboratory examinations (such as anti-dsDNA levels and complement levels), and SLE medications.

For the assessment of lupus nephritis, data on 24-hour urine protein (24hUP) levels, serum creatine levels, serum albumin levels, and hematuria were recorded. In accordance with the Aspreva Lupus Management Study,^[23]^ complete renal remission was defined as 24hUP < 0.5 g, normal urinary sediment, and serum creatine within 15% of the baseline value, and partial renal remission was defined as a 50% reduction in 24hUP, a 24hUP of < 3.5 g and serum creatine within 25% of the baseline value.

SLE disease activity was evaluated using Systemic Lupus Erythematosus Disease Activity Index (SLEDAI)-2K and the physician’s global assessment (PhGA) scores. Clinical response was defined as a reduction of ≥4 points in SLEDAI-2K with an increase of < 0.3 points in PhGA. Clinical remission on therapy was defined as an SLEDAI-2K score of 0 and a PhGA of < 0.5, with a maximum glucocorticoid dose of ≤5 mg/day (prednisone or equivalent).^[13]^ LLDAS was defined as follows: (1) an SLEDAI-2K score of ≤4 without scores for renal, central nervous system, serositis, vasculitis or constitutional components; (2) no increase in any component since the previous visit; (3) a PhGA score of ≤1; and (4) a glucocorticoid dose of ≤7.5 mg per day (prednisone or equivalent).^[14]^ The use of hydroxychloroquine and maintenance immunosuppressants was permitted under both definitions.

The primary endpoints were LN remission and SLE remission/LLDAS status. Secondary endpoints included changes in 24hUP levels and hematuria, changes in SLEDAI and PhGA scores, clinical response, serum complement levels, anti-dsDNA antibody titer, and glucocorticoids dosage.

### Statistical Analysis

Propensity score matching (PSM) was utilized to balance the baseline characteristics between the two groups. Independent variables included sex, age at baseline, SLE disease duration, SLEDAI score, and glucocorticoid dosage at baseline, with the use of sirolimus as the dependent variable. The propensity score was calculated using logistic regression, and a 1∶1 case-to-control ratio was applied. The optimal matching method was employed to minimize the Mahalanobis distance of the logit of the propensity score. The effectiveness of sirolimus versus MMF were compared between the two groups. Quantitative variables were described as means or medians and analyzed using Student’s t-test or the Wilcoxon signed ranks test, depending on their distribution. Categorical variables were described as counts and percentages and analyzed with χ2 test or Fisher’s exact test, as appropriate. A *P* value of < 0.05 was considered statistically significant. All analyses were performed using SPSS 23.0.0.0 (IBM Corp., Armonk, NY) and the results were visualized using GraphPad Prism version 8.0.2 (GraphPad Software, San Diego, CA).

## Results

### Baseline Characteristics

After PSM, 53 patients treated with MMF were matched to 53 patients treated with sirolimus. After matching, there was no significant differences in baseline demographic, clinical manifestations, laboratory parameters, and therapeutic features between the two groups. Renal biopsies were performed in 14 (26.4%) patients in sirolimus group and 13 (24.5%) patients in MMF group. The pathological classification of LN was predominantly type III/IV ± V in both group (9 patients in sirolimus group and 10 patients in MMF group). In sirolimus group, 13 patients were switched to sirolimus, and 16 patients had sirolimus added to their prior treatment (background immunosuppressants: 11 MMF, 3 AZA, 1 TAC, and 1 leflunomide). In MMF group, 8 patients were switched to MMF, and 5 patients had MMF added to their prior treatment (background immunosuppressants: 3 TAC, 1 AZA and 1 leflunomide). Other patients in both groups had not received any prior immunosuppressive agent and were initiated on MMF or sirolimus as monotherapy at baseline. Most patients in both groups (41/53 patients in sirolimus group and 47/53 patients in MMF group) were treated with hydroxychloroquine at baseline. No patients underwent glucocorticoid pulse therapy at baseline. In MMF group, 5 patients had hypertension and 1 patient had diabetes at baseline. In sirolimus group, 8 patients had hypertension and 2 patients had diabetes at baseline. Other baseline characteristics of patients in both groups were summarized in Table 1.

**Table 1:** Pre-PSM and post-PSM characteristics of the sirolimus and mycophenolate mofetil groups at baseline

	Pre-PSM			Post-PSM		
	Sirolimus (*N* = 63)	Mycophenolate mofetil (*N* = 622)	*P* value	Sirolimus (*N* = 53)	Mycophenolate mofetil (*N* = 53)	*P* value
Female, *n* (%)	55 (87.3%)	569 (91.5%)	0.267	46 (86.8%)	47 (88.7%)	0.767
Age (years)	35.4 ± 10.2	32.3 ± 11.8	0.01	34.9 ± 10.6	34.5 ± 11.4	0.679
Disease duration (months)	87.00 (25.00, 127.00)	29.00 (7.00, 82.00)	<0.001	87.00 (20.00, 123.00)	65.00 (14.00, 117.50)	0.246
SLEDAI-2K	7.00 (5.00, 12.00)	5.50 (2.00, 10.00)	0.004	8.00 (5.00, 12.00)	8.00 (5.00, 12.00)	0.556
PhGA	1.13 ± 0.53	1.23 ± 0.67	0.304	1.15 ± 0.53	1.18 ± 0.58	0.54
SLICC/ACR damage score	0.00 (0.00, 1.00)	0.00 (0.00, 1.00)	0.113	0.00 (0.00, 1.00)	0.00 (0.00, 1.00)	0.488
Lupus nephritis						
24hUP≥0.5 g, *n* (%)	36 (57.1%)	274 (44.1%)	0.047	33 (62.3%)	33 (62.3%)	1
24hUP level (g)	1.00 (0.48, 2.22)	0.98 (0.40, 2.20)	0.853	1.10 (0.54, 2.61)	1.05 (0.61, 1.84)	0.735
Hematuria, *n* (%)	23 (36.5%)	133 (21.4%)	0.006	19 (35.8%)	19 (35.8%)	1
Serum creatinine level (μmol/L)	73.9 ± 47.2	77.4 ± 66.1	0.819	75.4 ± 51.3	81.3 ± 63.5	0.976
Serum albumin level (g/L)	37.8 ± 4.9	36.3 ± 7.7	0.353	37.9 ± 5.2	35.3 ± 7.9	0.218
Laboratory parameters						
Elevated anti-dsDNA, *n* (%)	36 (57.1%)	277 (44.5%)	0.056	31 (58.5%)	31 (58.5%)	1
hypocomplementaemia, *n* (%)	42 (66.7%)	336 (54.0%)	0.054	35 (66.0%)	35 (66.0%)	1
C3 level (g/L)	0.674 ± 0.267	0.716 ± 0.303	0.293	0.690 ± 0.275	0.663 ± 0.305	0.641
C4 level (g/L)	0.105 (0.061, 0.147)	0.130 (0.074, 0.191)	0.033	0.117 (0.071, 0.158)	0.116 (0.071, 0.180)	0.823
GC dosage (mg/d)	10.0 (10.0, 30.0)	17.5 (10.0, 45.0)	0.003	12.5 (10.0, 30.0)	10.0 (10.0, 16.3)	0.218
Initial dosage of Mycophenolate mofetil (g/d) or sirolimus (mg/d)	NA	NA	NA	1.00 (1.00, 1.00)	1.50 (1.00, 1.50)	NA

PSM: propensity score matching, SLEDAI-2K: Systemic Lupus Erythematosus Disease Activity Index 2000, PhGA: physician’s global assessment, SLICC/ACR: Systemic Lupus International Collaborating Clinics / American College of Rheumatology, NPSLE: neuropsychiatric systemic lupus erythematosus, 24hUP: 24-hour urine protein,ANA: antinuclear antibody, anti-dsDNA: anti-double-stranded DNA, GC: glucocorticoid.

### Effectiveness of Sirolimus Versus MMF

**Figure 2:**
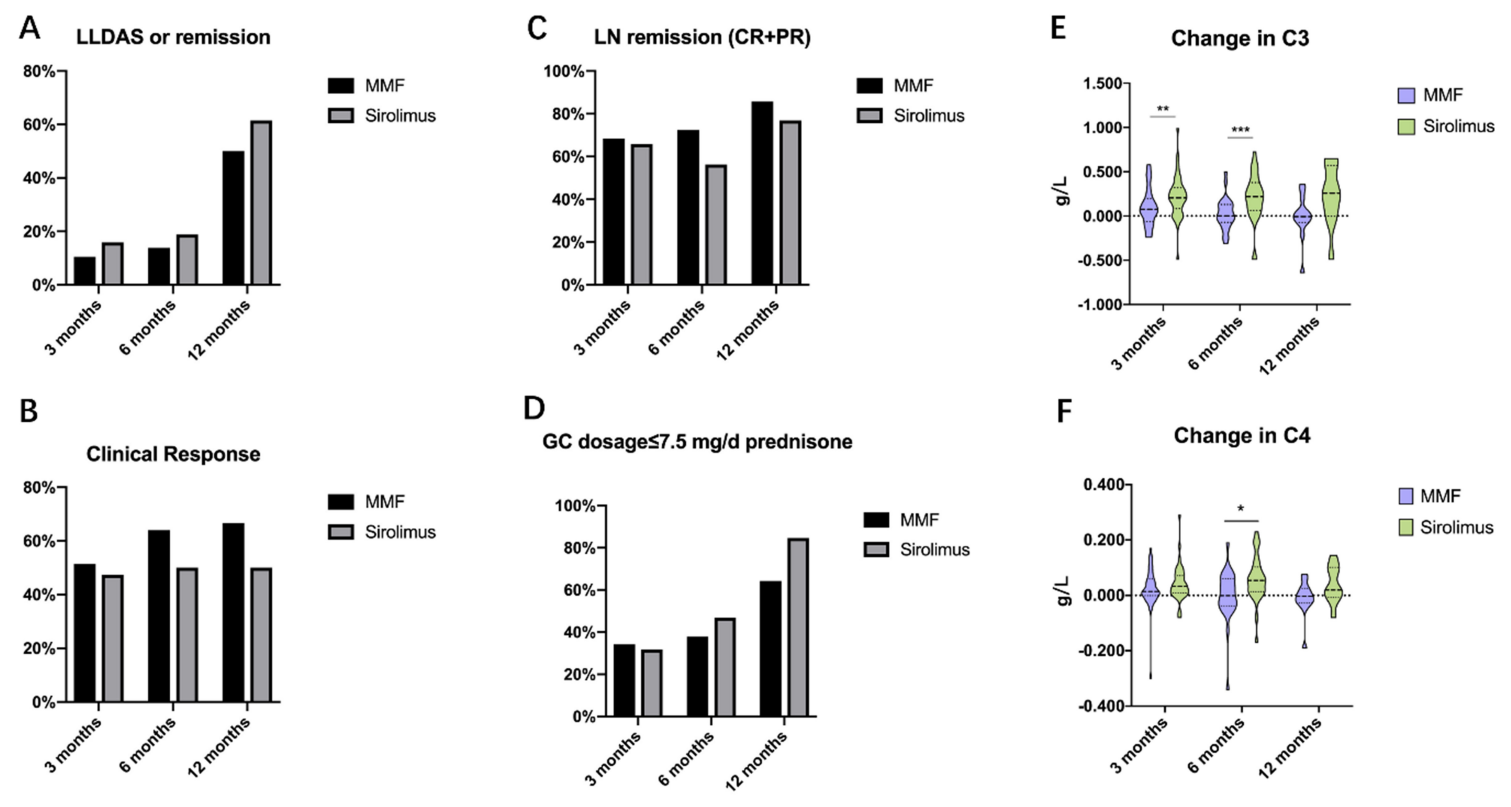
Clinical effectiveness of sirolimus versus MMF in treating lupus nephritis (LN) at 3-, 6-, and 12-month. MMF: mycophenolate mofetil, LLDAS: lupus low disease activity state, GC: glucocorticoid, CR: complete remission, PR: partial remission.*P < 0.05; **P < 0.01; ***P < 0.001.

Data on the effectiveness of sirolimus versus MMF at 3, 6, and 12 months were presented in Table 2 and Figure 2. Both sirolimus and MMF demonstrated good efficacy in treating LN. The proportions of patients achieving complete or partial renal remission was similar between the two groups (65.9% *vs*. 68.4% at 3 months, 56.3% *vs*. 72.4% at 6 months, and 76.9% *vs*. 85.7% at 12 months, *P* = 0.809, 0.189, and 0.557, respectively), as well as the prevalence of hematuria (27.3% *vs*. 31.6% at 3 months, 31.3% *vs*. 13.8% at 6 months, and 15.4% *vs*. 7.1% at 12 months, *P* = 0.669, 0.105, and 0.496, respectively), and the change in 24hUP level (-0.960 (-2.083, 0.115) *vs*. -0.287 (-0.900, -0.002) at 3 months, -0.220 (-1.540, 0.270) *vs*. -0.669 (-2.085, -0.475) at 6 months, and-1.470 (-4.410, 0.150) *vs*. -0.675 (-1.433, 1.750) at 12 months, *P* = 0.284, 0.121, and 0.440, respectively).

**Table 2:** Efficacy of sirolimus vs. mycophenolate mofetil (MMF) in treating patients with lupus nephritis at 3-, 6-, and 12-month

	3 months			6 months			12 months		
	Sirolimus	MMF	*P* value	Sirolimus	MMF	*P* value	Sirolimus	MMF	*P* value
SLEDAI-2K change	-2.00 (-6.00, 0.00)	-4.00 (-8.00, 0.00)	0.228	-2.00 (-6.00, 0.00)	-4.00 (-8.00, -1.00)	0.141	-2.00 (-6.00,-0.50)	-4.00 (-6.00, -1.50)	0.532
	-0.200	-0.250		-0.200	-0.200		-0.400	-0.350	
PhGA change	(-0.600, 0.000)	(-0.650, 0.200)	0.928	(-0.800, 0.100)	(-0.750, 0.200)	0.743	(-0.650,-0.025)	(-0.775, 0.150)	0.908
	47.4%	51.4%		50.0%	64.0%				
Clinical response	(18/38)	(18/35)	0.729	(13/26)	(16/25)	0.313	50.0% (5/10)	66.7% (8/12)	0.429
LLDAS/remission	15.9% (7/44)	10.5% (4/38)	0.476	18.8% (6/32)	13.8% (4/29)	0.602	61.5% (8/13)	50.0% (7/14)	0.547
Lupus nephritis									
Remission (CR+PR)	65.9%	68.4%	0.809	56.3%	72.4%	0.189	76.9%	85.7%	0.557
	(21+8/44)	(24+2/38)		(17+1/32)	(20+1/29)		(8+2/13)	(12+0/14)	
	-0.960	-0.287		-0.220	-0.669		-1.470	-0.675	
24hUP level change (g)	(-2.083, 0.115)	(-0.900, -0.002)	0.284	(-1.540, 0.270)	(-2.085, -0.475)	0.121	(-4.410, 0.150)	(-1.433, 1.750)	0.440
	27.3%	31.6%		31.3%	13.8%				
Hematuria	(12/44)	(12/38)	0.669	(10/32)	(4/29)	0.105	15.4% (2/13)	7.1% (1/14)	0.496
Laboratory parameters									
Normalised anti-dsDNA	41.7%	66.7%	0.082	28.6%	60.0%	0.059	50.0% (3/6)	42.9% (3/7)	0.797
	(10/24)	(16/24)		(6/21)	(9/15)				
Recovered hypocomplementaemia	74.1%	41.7%	0.019	75.0%	30.0%	0.003	71.4% (5/7)	0% (0/8)	0.003
	(20/27)	(10/24)		(18/24)	(6/20)				
	0.206 (0.084, 0.321)	0.075		0.219	0.001		0.259	-0.007	
Change in C3 (g/L)		(-0.063, 0.196)	0.008	(0.062, 0.378)	(-0.072, 0.130)	0.001	(-0.003, 0.570)	(-0.074, 0.125)	0.063
		0.014		0.054	-0.001		0.020	-0.003	
Change in C4 (g/L)	0.033 (0.009, 0.071)	(-0.001, 0.060)	0.250	(0.014, 0.103)	(-0.038, 0.060)	0.011	(-0.008, 0.101)	(-0.028, 0.026)	0.121
Use of GC									
							-5.00	-6.25	
Change in GC dosage (mg/d)	0.00 (-13.75, 0.00)	0.00 (-5.00, 0.00)	0.174	0.00 (-10.63, 0.00)	0.00 (-6.25, 0.00)	0.771	(-22.50, 0.00)	(-12.50,-1.88)	0.961
GC dosage≤7.5 mg/d prednisone	31.8%	34.2%		46.9%	37.9%		84.6%		
	(14/44)	(13/38)	0.818	(15/32)	(11/29)	0.481	(11/13)	64.3% (9/14)	0.228

SLEDAI-2K: Systemic Lupus Erythematosus Disease Activity Index 2000, PhGA: physician’s global assessment, LLDAS: lupus low disease activity state, CR: complete remission, PR: partial remission, 24hUP: 24-hour urine protein, anti-dsDNA: anti-double-stranded DNA, GC: glucocorticoid.

Improvement in SLE disease activity were comparable between the two groups. Changes in SLEDAI-2K score (-2.00 (-6.00, 0.00) *vs*. -4.00 (-8.00, 0.00) at 3 months, -2.00 (-6.00, 0.00) *vs*. -4.00 (-8.00, -1.00) at 6 months, and-2.00 (-6.00, -0.50) *vs*. -4.00 (-6.00, -1.50) at 12 months, *P* = 0.228, 0.141, and 0.532, respectively) and PhGA score (-0.200 (-0.600, 0.000) *vs*. -0.250 (-0.650, 0.200) at 3 months, -0.200 (-0.800, 0.100) *vs*. -0.200 (-0.750, 0.200) at 6 months, and-0.400 (-0.650, -0.025) *vs*. -0.350 (-0.775, 0.150) at 12 months, P= 0.928, 0.743, and 0.908, respectively) showed no significant differences. Additionally, the proportions of patients achieving clinical response (47.4% *vs*. 51.4% at 3 months, 50.0% *vs*. 64.0% at 6 months, and 50.0% *vs*. 66.7% at 12 months, *P* = 0.729, 0.313, and 0.429, respectively), LLDAS or remission (15.9% *vs*. 10.5% at 3 months, 18.8% *vs*. 13.8% at 6 months, and 61.5% *vs*. 50.0% at 12 months, *P* = 0.476, 0.602, and 0.547, respectively) were similar between the two groups.

Regarding serological activity, the sirolimus group demonstrated significantly greater improvement in hypocomplementemia compared to MMF group, with higher rates of normalized complement levels at all follow-up intervals (74.1% *vs*. 41.7% at 3 months, 75.0% *vs*. 30.0% at 6 months, and 71.4% *vs*. 0% at 12 months, *P* = 0.019, 0.003 and 0.003, respectively). Significant differences were also observed in change of serum C3 level (0.206 (0.084, 0.321) *vs*. 0.075 (-0.063, 0.196) at 3 months, 0.219 (0.062, 0.378) *vs*. 0.001 (-0.072, 0.130) at 6 months, and 0.259 (-0.003, 0.570) *vs*. -0.007 (-0.074, 0.125) at 12 months, *P* = 0.008, 0.001, and 0.063, respectively) and C4 level (0.033 (0.009, 0.071) *vs*. 0.014 (-0.001, 0.060) at 3 months, 0.054 (0.014, 0.103) *vs*. -0.001 (-0.038, 0.060) at 6 months, and 0.020 (-0.008, 0.101) *vs*. -0.003 (-0.028, 0.026) at 12 months, *P* = 0.250, 0.011, and 0.121, respectively). No significant differences were observed in the proportion of patients achieving normalized anti-dsDNA antibody between the two groups (41.7% *vs*. 66.7% at 3 months, 28.6% *vs*. 60.0% at 6 months, and 50.0% *vs*. 42.9% at 12 months, *P* = 0.082, 0.059, and 0.797, respectively).

The change in glucocorticoid (GC) dosage [0.00 (-13.75, 0.00) *vs*. 0.00 (-5.00, 0.00) at 3 months, 0.00 (-10.63, 0.00) *vs*. 0.00 (-6.25, 0.00) at 6 months, and -5.00 (-22.50, 0.00) *vs*. -6.25 (-12.50, -1.88) at 12 months, mg/d, *P* = 0.174, 0.771, and 0.961, respectively] and the proportions of patients achieving prednisone doses ≤7.5 mg/day (31.8% *vs*. 34.2% at 3 months, 46.9% *vs*. 37.9% at 6 months, and 84.6% *vs*. 64.3% at 12 months, *P* = 0.818, 0.481 and 0.228, respectively) were not significantly different between the two groups.

During the follow-up period, none of the 106 patients in either group switched therapies or required additional immunosuppressants or biologic agents such as belimumab or rituximab.

### Safety of Sirolimus and MMF

The safety profiles of sirolimus and MMF were evaluated during the study. In sirolimus group, a total of 10 adverse events were recorded, including 3 cases of mild renal insufficiency, 2 cases of mild infections, 1 case of mild haemocytopaenia, 1 case of facial edema, 1 case of skin rash, 1 case of alopecia, and 1 case of menstruation changes. In MMF group, 1 adverse event of mild infection was recorded. No severe adverse events occurred in either group.

## Discussion

This real-world, prospective, open-label cohort study compared the effectiveness and safety of sirolimus with MMF for the treatment of LN. To the best of our knowledge, this is the first prospective study to compare sirolimus against SoC therapies and provide treat-to-target (T2T) evidence in LN management. Sirolimus demonstrated similar clinical effectiveness and steroid-sparing effect to MMF, and better effectiveness in serological improvement. In comparison to the MMF group, the sirolimus group exhibited no significant differences in rates of LN remission (65.9% *vs*. 68.4%, 56.3% *vs*. 72.4%, and 76.9% *vs*. 85.7% at three follow-up visits) and LLDAS/remission (15.9% *vs*. 10.9%, 18.8% *vs*. 13.8%, and 61.5% *vs*. 50.0% at three follow-up visits). Significantly greater improvements in complement levels were observed in sirolimus group, especially at 6-month follow-up (75% *vs*. 30%, *P* < 0.01). These outcomes are consistent with prior CSTAR studies^[24,25]^ and other cohorts,^[17,18]^ reinforcing sirolimus as a well-tolerated and effective option for LN.

Sirolimus, also named as rapamycin, is a mTOR inhibitor which has been successfully used for the prevention of graft-versus-host disease. Activation of mTOR signaling has been observed in SLE patients,^[16]^ and this pathway may contribute to various pathogenesis mechanisms of SLE, including induction of interleukin (IL)-4 production and necrotic death of double-negative T cells,^[26]^ increasing interferon α (IFNα) production,^[27]^ promotion of BAFF-stimulated cell proliferation and survival,^[28]^ promotion of Th17 cell differentiation,^[29]^ and inhibition of CD4^+^ T cell differentiation into Treg cells.^[30]^

The efficacy of sirolimus in treating autoimmune diseases and SLE has been reported in several studies.^[24,25,31]^ A multicenter, prospective clinical trial involving 30 patients demonstrated the effectiveness of sirolimus in relapsed/refractory autoimmune cytopenia.^[32]^ A single-center, single-arm, phase II study conducted with 20 patients from the CSTAR registry highlighted the efficacy and safety of sirolimus in treating refractory connective tissue disease-related thrombocytopenia. ^[33]^ A real-world study enrolled 49 SLE patients from CSTAR registry found that sirolimus significantly improved skin rash, arthritis, and thrombocytopenia.^[25]^ Eriksson *et al*. had reported that sirolimus was effective in SLE patients with musculoskeletal manifestations, such as arthritis and tendinitis, in a retrospective Swedish cohort involving 27 SLE patients with long-term follow-up (mean time: 47.1 months).^[34]^ Despite these promising findings, the role of sirolimus in LN still warrants further investigation.

Regarding LN, animal experiments and in vitro evidence have demonstrated that mTOR inhibition by sirolimus can ameliorate the manifestation of LN.^[35, 36, 37]^ In LN patients, renal mTOR complex 1 activation has been proposed as a biomarker for disease activity and clinical prognosis.^[38]^ The mTOR activation might affect the pathogenesis of LN through various mechanisms, including the activation of Th17 cells and IL-17 signaling pathway,^[37,39]^ activation of Th1 cells and suppression of dendritic cells (DC),^[35]^ promotion of intra-renal expression of monocyte chemoattractant protein-1 (MCP-1),^[36]^ and the dysregulation of autophagy not only in T and B cells,^[40]^ but also in renal resident cells such as podocytes.^[41]^

Despite these mechanistic insights, clinical evidence supporting sirolimus use in LN remains limited. Case reports indicated that sirolimus may be effective in patients with LN refractory to conventional immunosuppressants, such as CYC, MMF, tacrolimus or cyclosporin A.^[42,43]^ Retrospective studies have also highlighted its potential in LN. A study of 16 LN patients treated with sirolimus for an average duration of 45.3 months^[18]^ reported significant improvement in proteinuria, with only one patient experiencing renal flare. Another cohort of 12 LN patients revealed the improvements in proteinuria and hematuria following long-term sirolimus treatment.^[17]^ Previous CSTAR studies involving SLE patients with various manifestations have also suggested the potential effectiveness of sirolimus in achieving LN remission.^[24,25]^ However, no prospective controlled studies have specifically evaluated the effectiveness of sirolimus in LN. The first CSTAR study comparing sirolimus with conventional immunosuppressants in SLE found sirolimus to be as effective as tacrolimus.^[24]^ Consistent with these findings, our study provides further evidence by comparing sirolimus with SoCs treatments in LN.

Our study demonstrated that sirolimus exhibits comparable efficacy to MMF, the SoCs for LN according to guideline.^[4]^ No significant differences were observed between the two groups in terms of the primary endpoint and main clinical secondary endpoints related to SLE and LN activity. In recent years, the implementation of T2T strategy for SLE and LN, which was proven in improving clinical outcomes, has become a clinical reality.^[44]^ To provide robust T2T evidence, our study specifically excluded patients who had already achieved LLDAS or remission at baseline. The results were encouraging, as sirolimus and MMF showed no significant differences in clinical response, LLDAS/remission rates, or renal remission. The equivalence in efficacy, particularly for T2T endpoints not previously reported, positions sirolimus as a promising novel treatment option for LN. The 2023 updated European Alliance of Associations for Rheumatology (EULAR) recommendation for SLE highlighted the combination therapy with belimumab (either with CYC or MMF) or calcineurin inhibitors (especially voclosporin or TAC, combined with MMF), in the initial therapy of active LN.^[45]^ Notably, in our study, 16 patients received sirolimus as an add-on therapy with background immunosuppressants (primarily MMF), suggesting that sirolimus may also serve as a novel treatment option for combination therapy in LN.

Sirolimus has demonstrated good effect on the improvement of serological parameters in SLE and LN. Previous studies from the CSTAR registry and other cohorts have shown that sirolimus significantly improves hypocomplementemia in SLE and LN patients.^[17,18,25]^ Consistent with these findings, our study also observed notable improvements in hypocomplementemia. The CSTAR cohort ^[25]^ and US-based SLE cohort^[17]^ reported significant reductions in anti-dsDNA titers following sirolimus treatment. However, in our study, no significant differences in anti-dsDNA changes were observed between the two groups among LN patients, aligning with the results from other cohorts involving LN patients.^[17,18]^

Sirolimus has also shown promising effect on steroid-sparing in a single-arm, open-label, phase 1/2 trial involving 43 SLE patients.^[19]^ In this study, the mean daily dose of prednisone required to control disease activity decreased from 23.7 mg to 7.2 mg after 12 months of sirolimus treatment. Similarly, our study observed a trend toward steroid dose reduction, particularly at 12-month follow-up. However, no significant difference was noted between the two groups. This may be attributed to the relatively low initial glucocorticoid dosage in our study and the fact that MMF, a classic immunosuppressant, is also known to have steroid-sparing effects in LN.

There were several limitations in our study. Firstly, as a real-world study with retrospective analysis of prospectively collected data, there were inherent differences in baseline characteristics, disease conditions, and previous background immunosuppressants between the two groups. Although we utilized PSM method to minimize the selection bias, some residual confounding might still have influenced the results. Secondly, the proportion of LN patients who underwent renal biopsy in CSTAR cohort was relatively low (16.6% in previous report,^[46]^ 25.5% in our study). It might be attributed to the fact that the CSTAR cohort was primarily established by rheumatologist rather than nephrologists, who have more experience with renal biopsy. Future efforts should focus on improving the implementation of renal biopsy for active LN patients. Third, our study lacked longterm follow-up data for sirolimus in LN, as reported in earlier retrospective study.^[18]^ The number of patients with 12-month or longer follow-up data was limited, which may introduce bias in the data analyses. Therefore, further high-quality, randomized, double-blind, controlled clinical trials involving more patients with renal biopsy data and extended follow-up duration are needed to establish the efficacy and safety of sirolimus in LN patients.

In conclusion, this study represented the first comparison of the effectiveness and safety of sirolimus versus MMF, the SoC recommended by LN guidelines, in the treatment of LN. It also provides T2T evidence supporting the use of sirolimus in LN. Sirolimus and MMF demonstrated similar effectiveness in achieving disease activity remission and glucocorticoid dosage tapering. Moreover, sirolimus exhibited greater effectiveness in improving serological parameters compared to MMF. Importantly, sirolimus was well tolerated among LN patients. These findings suggest that sirolimus is a promising novel therapeutic option for LN. However, further high-quality studies are needed to confirm the efficacy and safety of sirolimus for LN treatment.
